# Intrinsically chiral exciton polaritons in an atomically-thin semiconductor

**DOI:** 10.1038/s41467-026-70875-5

**Published:** 2026-03-23

**Authors:** M. J. Wurdack, I. Iorsh, S. Vavreckova, T. Bucher, M. Król, Z. Fedorova, E. Estrecho, D. Ilin, S. Klimmer, L. P. L. Mawlong, H. Deng, Q. Song, T. van der Laan, G. Soavi, T. Pertsch, F. Eilenberger, I. Staude, Y. Kivshar, E. A. Ostrovskaya

**Affiliations:** 1 Institute of Solid State Physics, Friedrich Schiller University Jena, Jena, Germany; 2 Department of Quantum Science and Technology, Research School of Physics, The Australian National University, Canberra, ACT Australia; 3 Institute of Applied Physics, Friedrich Schiller University Jena, Jena, Germany; 4 Abbe Center of Photonics, Friedrich Schiller University Jena, Jena, Germany; 5 Department of Chemical Engineering, Stanford University, Stanford, CA USA; 6 Department of Physics, Engineering Physics and Astronomy, Queen’s University, Kingston, ON Canada; 7 School of Mathematical and Physical Sciences, University of Technology Sydney, Ultimo, NSW Australia; 8 Manufacturing, CSIRO, West Lindfield, Sydney, NSW Australia; 9 Harbin Institute of Technology, Shenzhen, China; 10 Fraunhofer-Institute for Applied Optics and Precision Engineering IOF, Jena, Germany; 11 Nonlinear Physics Center, Research School of Physics, Australian National University, Canberra, ACT Australia

**Keywords:** Polaritons, Two-dimensional materials, Photonic crystals

## Abstract

Photonic bound states in the continuum (BICs) have emerged as a versatile tool for enhancing light-matter interactions by strongly confining light fields. Chiral BICs are photonic resonances with a high degree of circular polarisation, which hold great promise for spin-selective applications in quantum optics and nanophotonics. Here, we demonstrate a novel application of a chiral BIC for inducing strong coupling between the circularly polarised photons and spin-polarised (valley) excitons (bound electron-hole pairs) in atomically-thin transition metal dichalcogenide crystals (TMDCs). By placing monolayer WS_2_ onto the BIC-hosting metasurface, we observe the formation of intrinsically chiral, valley-selective exciton polaritons, evidenced by circularly polarised photoluminescence (PL) at two distinct energy levels. The PL intensity and degree of circular polarisation of polaritons exceed those of uncoupled excitons in our structure by an order of magnitude. Our microscopic model shows that this enhancement is due to folding of the Brillouin zone creating a direct emission path for high-momenta polaritonic states far outside the light cone, thereby providing a shortcut to thermalisation (energy relaxation) and suppressing depolarisation. Moreover, while the polarisation of the upper polariton is determined by the valley excitons, the lower polariton behaves like an intrinsic chiral emitter with its polarisation fixed by the BIC. Therefore, the spin alignment of the upper and lower polaritons (*↑↓* and *↑↑*) can be controlled by *σ*^+^ and *σ*^−^ circularly polarised optical excitation, respectively. Our work introduces a new type of chiral light-matter quasi-particles in atomically-thin semiconductors and provides an insight into their energy relaxation dynamics.

## Abstract (web summary)

The authors observe strong coupling between valley excitons in a monolayer WS2 and intrinsically chiral photons shaped by a metasurface. The polaritons display enhanced chiral emission with their spin configuration controlled by the light source.

## Introduction

**Fig. 1:**
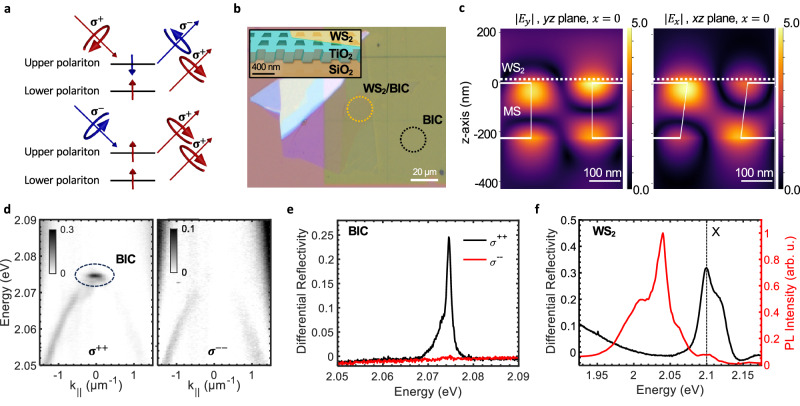
Optical properties of the WS_2_/BIC heterostructure. **a** Schematics of the spin configuration and emission properties of upper and lower polaritons under (top) *σ*^+^ and (bottom) *σ*^−^ polarised optical excitation. **b** Microscope image and (inset) schematic illustration of the heterostructure consisting of the metasurface governed by chiral BICs^4^ and the monolayer WS_2_. The measured areas are marked with yellow and black circles. **c** Electric field distribution of the metasurface at the BIC resonance *λ* = 597 nm in its linear polarisation bases (left) $\left|{E}_{y}\right|$ and (right) $\left|{E}_{x}\right|$ in the yz- and xz-plane, respectively. **d** Angle-resolved differential reflectivity spectra of the metasurface next to the position of the monolayer (black circle in **b**) of (left) *σ*^+^ and (right) *σ*^−^ polarised white light, where the reflected light is filtered in its *σ*^+^ and *σ*^−^ polarisaton bases, respectively. The response of the chiral BIC is marked with a black dashed circle. **e** Polarisation resolved reflectivity spectra of panel **d** plotted along *k* = 0. **f** Reflectivity and photoluminescence spectrum at the position of the monolayer WS_2_ (yellow circle in **b**) at *T* ≈ 4K, measured at large momenta (*k*_∣∣_ ≈ 4.5 μm^−1^, see ‘Methods’) to avoid the influence of the BIC.

Chiral photonic and electronic states are the building blocks for spin-based applications, which include quantum computing and communication, spintronics, and spin-selective sensing. Time-reversal symmetry breaking, parity-time-symmetry breaking or band gap inversion can induce such chiral states^1–7^. Breaking the symmetry of a metasurface hosting photonic bound states in the continuum (BICs)^8–10^ can generate chiral BICs that interact with either *σ*^+^ or *σ*^−^ circularly polarised light, i.e. by separating their *σ*^+^ and *σ*^−^ polarisation bases in parameter space^4,5,11^. Similarly, in semiconducting atomically thin (monolayer) transition-metal dichalcogenide crystals (TMDCs), broken inversion symmetry combined with strong spin-orbit coupling leads to energetically degenerate band edges of the Brillouin zone with alternating spins, where spin-polarised bound electron-hole pairs (excitons) form in the K and K′ valleys (see Fig. 1a)^2,12,13^. Thus, *σ*^+^ and *σ*^−^ polarised light can selectively probe K and K′ valley excitons, respectively, which is facilitated by their high oscillator strength and strong photoluminescence (PL)^13–17^. However, experimentally measured degrees of circular polarisation and valley coherence in TMDCs are often much lower than 1^15^, mostly due to inelastic scattering events, e.g. with phonons and disorder^17^, which strongly limits their potential applications. Scattering can be suppressed by forming exciton polaritons (polaritons herein) in the strong light-matter coupling regime^18^, i.e. by placing a TMDC monolayer in a strongly confined light field of a microcavity or a photonic crystal^19^. While polaritons in monolayer TMDCs are well studied^19,20^, these studies are limited to achiral photonic structures, where spin-selective behaviour is mainly inherited by the valley-degenerate excitons^21^.

Here, we demonstrate the formation of intrinsically chiral, valley-selective polaritons in a monolayer WS_2_ placed on a metasurface governed by chiral BICs^4^. BICs were recently introduced as versatile photonic resonances for achieving polariton formation in a range of materials^22–24^. The choice of material in this work, monolayer WS_2_, is motivated by its spin-valley physics^25^ and the ability to reach a strong light-matter coupling regime with its excitons in a wide temperature range, including room temperature^18,23,26–28^. We perform experimental and theoretical reflectivity and PL studies showing that, in the strong light-matter coupling regime, diffraction overcomes thermalisation-induced losses and depolarisation, which leads to massively enhanced circularly polarised PL of both the upper and lower polariton branches. Remarkably, lower and upper polaritons can have parallel (*↑↑*) or anti-parallel (*↑↓*) spin-configurations, which can be switched with *σ*^−^ or *σ*^+^ polarised excitation, respectively, leading to co- or anti-polarised PL from the two energy levels (see Fig. 1a). This is because both photonic and excitonic constituents of the polaritons contribute to the spin-configurations, with the photonic (excitonic) contribution being stronger for the lower (upper) polariton. Thus, the lower polariton inherits most of its properties from the chiral BIC, and only interacts with and emits *σ*^+^ polarised light^4^. In contrast, the upper polariton shows rotating dipole behaviour as inherited from the exciton, reversing the polarisation of the exciting light^29^. Additionally, the spin degree of freedom of valley excitons also manifests itself in the lower polariton, where the PL is the strongest when the exciton spin is aligned with that of the BIC. As such, combining the spin-selective light and matter states in a hybrid polaritonic state offers a novel pathway for the control and manipulation of chiral light emission.

## Results

The structure investigated in this work consists of an exfoliated monolayer WS_2_ placed on a slant-perturbed metasurface, i.e. a TiO_2_ layer deposited on SiO_2_ and patterned with a square array of trapezoid nanoholes (see Fig. 1b, SI and ‘Methods’)^4^. Good spatial overlap between the photonic near-field mode of the metasurface and the monolayer, confirmed by the calculation of the electric field distribution (see Figs. 1c and SI), enables the coupling between the photonic mode and the exciton in the monolayer. The sample is illuminated by either a polarised tungsten-halogen white light source or a continuous-wave (CW) laser tuned to the WS_2_ bandgap and its reflectivity and PL properties are studied in the reflection geometry at cryogenic temperatures (*T* ≈ 4K) (see ‘Methods’). Area-dependent reflectivity measurements show that, on the spatial scales relevant to our white light spot and monolayer size, the hole count and fabrication accuracy are sufficient to provide a high-Q resonance (see SI).

### Spectral features of the chiral BIC and monolayer WS_2_

The measured angle-resolved reflectivity spectrum next to the position of the monolayer (see Fig. 1b) shows a discrete mode, which only interacts with *σ*^+^ polarised white light at *k*_∣∣_ = 0 and *E* ≈ 2.075 eV (see Fig. 1d, e), where *k*_||_ is the momentum in the plane of the monolayer. In particular, this mode disappears when the sample is illuminated by *σ*^−^ polarised light. This feature marks the optical response of the highly polarisation-selective chiral BIC with a Q-factor of ~1500. Henceforth, *σ*^*ir*^ convention indicates polarisations of the incoming (*i*) and reflected (*r*) light. The optical response of the monolayer was characterised via white light reflectivity and PL spectroscopy at large momenta (*k*_∣∣_ ≈ 4.5 μm^−1^) to avoid the influence of the BIC signal prevalent at *k*_∣∣_ = 0 (see ‘Methods’). The reflectivity and PL spectra in Fig. 1f show strong excitonic (X) absorption and weak PL emission at *E* ≈ 2.1 eV^16^, respectively. While at liquid Helium temperatures the PL of dark-type monolayer WS_2_ mostly stems from charged and multi-body complexes, e.g. at ~2.04 and ~2.06 eV^25,30–36^, the prominent exciton resonance at ~2.1 eV in the reflectivity spectrum^16^, which coincides with the weak exciton PL peak at the same energy, enables strong light-matter interactions and formation of exciton polaritons in this material^28^.

### Optical response in the strong light-matter coupling regime

**Fig. 2:**
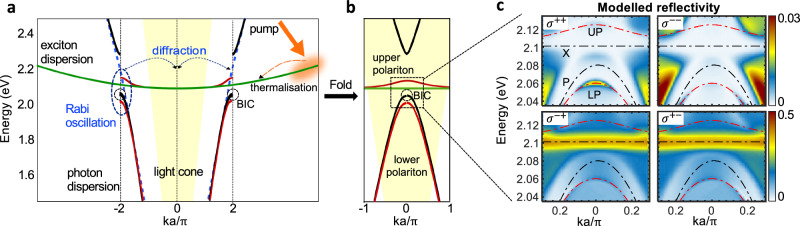
Model of exciton-photon coupling. **a** Band structure of the (black) relevant photonic, (green) excitonic and (red) polaritonic modes of the sample in the strong exciton-photon coupling regime, and schematic illustration of relaxation and diffraction processes that lead to photoluminescence. **b** Folded Brillouin zone of the photonic components, showing that both (green) excitons and (red) polaritons coexist within the light cone. The light cone of our experimental setup (see Methods) is shaded in yellow and the location of the relevant chiral BIC (*σ*^+^) on the photonic dispersion is marked with black dashed circles in (**a**, **b**). **c** Modelled white light reflectivity in the far field, which is dominated by the polariton response (red dashed line) at the native polarisation of the chiral BIC (*σ*^+^), and by the exciton response (X) when the polarisation of the reflected light is opposite to the polarisation of the incoming light (i.e. *σ*^+−/−+^). The polariton (UP/LP) and uncoupled exciton/photon (X/P) dispersions are marked as red and black dashed lines, respectively.

To describe the salient features of the optical response in the strong light-matter coupling regime, we calculate the dispersion curves of the relevant photonic, excitonic and polaritonic modes (see Fig. 2a, b). The periodic structure of the metasurface creates a photonic band gap at *ka*/*π* = ±2 (here *k*_∣∣_ = ±18.2 μm^−1^ with *a* = 340 nm, see ‘Methods’). The BIC is located at the energy maximum of the lower band, which is close to the exciton energy in monolayer WS_2_. With sufficiently large energy-exchange interactions, i.e. Rabi oscillation frequencies, level repulsion occurs leading to formation of the upper and the lower polariton branches (see red lines in Fig. 2a). Folding of the Brillouin zone of the metasurface projects the dispersive branches from high-momentum states far outside the light cone of our experimental setup to low-momentum states, e.g., *ka*/*π* = ±2 to *ka*/*π* = 0, making the polariton branches visible within the light cone (see Fig. 2b). As such, both excitons (unfolded) and polariton branches (folded) coexist within the light cone. This is in contrast to polaritons in optical microcavities, where in the strong exciton-photon coupling regime the uncoupled excitons are only present far outside of the light cone^37,38^.

When calculating the optical response (reflectivity) of the sample illuminated by circularly polarised white light (see ‘Methods’ and Fig. 2c), we find that the lower polariton behaves similarly to the chiral BIC mode, with the reflectivity signal present at *σ*^++^ and vanishing at *σ*^−−^ (cf. Figs. 1d and 2c, top panels). In contrast, the excitons, which are modelled as rotating dipoles, are responsible for the strong reflected signal with the polarisation opposite to that of the illumination *σ*^+−/−+^, which is similar to light reflection at a metallic surface^29,39^. This can be explained with our band structure (see Fig. 2a), which shows coexistence of excitons and polaritons within the light cone.

### Amplification of polariton emission

**Fig. 3:**
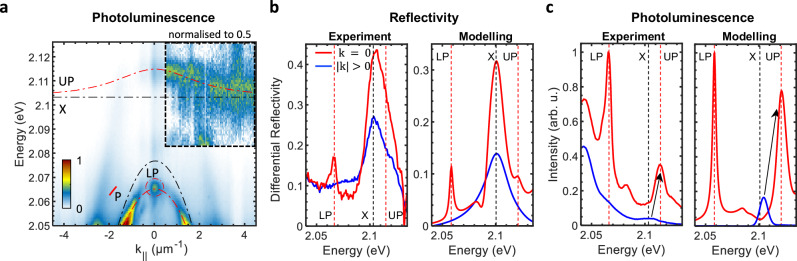
Reflectivity and photoluminescence spectra. **a** Experimental angle-resolved photoluminescence spectrum, with the fitted (LP) lower and (UP) upper polariton branches (red dashed lines), and the uncoupled (X) exciton and (P) photon dispersions (black dashed lines). The part of the upper polariton dispersion inheriting the chiral BIC properties is marked with a red dashed circle. The data in the inset are normalised to 0.5 for each *k*_∣∣_ value to visualise the peak positions of the low-intensity upper polariton emission. **b**, **c** Experimental and theoretical reflectivity and photoluminescence spectra at (red) *k*_∣∣_ = 0 and (blue) *k*_∣∣_ ≈ 4.5 μm^−1^, respectively. The arrows in (**c**) illustrate the order of magnitude enhancement of the PL intensities of the upper polariton between the lower energy state at *k*_∣∣_ ≈ 4.5 μm^−1^ and the higher energy state at *k*_∣∣_ = 0.

To verify our model experimentally, we performed angle-resolved white light reflectivity and PL spectroscopy at the position of the monolayer on the sample (see Fig. 1b). This was done by illuminating the sample with either a white light source or with a 561 nm (2.21 eV) cw-laser, which lies within the band gap of WS_2_ (*E*_*g*_ ≈ 2.4 eV^13^) and quasi-resonantly excites excitons at large momenta. Figure 3a shows the angle-resolved PL intensity of the structure with the peak energies well reproduced by two coupled oscillator model describing the lower/upper polariton eigenvalues (LP/UP) emerging in the system of a photon (P) strongly coupled to an exciton (X): ${E}_{{{\rm{LP/UP}}}}=\frac{1}{2}[{E}_{{{\rm{X}}}}+{E}_{{{\rm{P}}}}\pm \sqrt{{(2\hslash \Omega )}^{2}+{\delta }^{2}}]$^37^, with the exciton-photon energy detuning *δ* = *E*_X_ − *E*_P_ = 26.4 meV, and the Rabi splitting 2*ℏΩ* = 42.0 meV. The excitonic (∣*X*∣^2^) and photonic fractions (∣*C*∣^2^) of polaritons can be described with the corresponding Hopfield coefficients: $| {X}_{{{\rm{UP/LP}}}}{| }^{2}=\frac{1}{2}\left[1\pm \delta /\sqrt{{\delta }^{2}+4{\hslash }^{2}{\Omega }^{2}}\right]$, ∣*C*_UP/LP_∣^2^ = 1 − ∣*X*_UP/LP_∣^2^ ^37^, which in our system amount to ∣*X*_UP/LP_∣^2^ ≈ 0.76/0.24 at *k*_∣∣_ = 0. The localised emission peak at *k*_∣∣_ = 0 and *E* ≈ 2.066 eV, reminiscent of the chiral BIC response at *E* ≈ 2.077 eV(see Fig. 1d), originates from the more photonic LP. The emission above 2.1 eV corresponds to the more excitonic UP. The UP disperison near *k*_∣∣_ = 0, when folded as shown in Fig. 2a, b, is strongly modified by level repulsion in proximity to the BIC state causing anti-crossing with the exciton level (X). This confirms the strong coupling regime between the WS_2_ excitons and the chiral photonic BIC.

The PL signal, which is dominated by polaritonic states, is in stark contrast to the reflectivity spectrum (see Fig. 3b), which, as predicted by our model (see Fig. 2c), is strongly dominated by the exciton mode. Both high- and low-momenta spectra are well reproduced by our calculations (see polarisation-resolved spectra in the SI), with the sharp LP peak only present at low momenta (around *k*_||_ = 0). This agreement confirms the accuracy of our model and that both excitons and polaritons coexist within the light cone.

Remarkably, the exciton peak in the PL is strongly suppressed (see Fig. 3a, c). In particular, the PL intensity of polaritons at *k*_∣∣_ = 0 is an order of magnitude larger than that of the energetically lower more excitonic emission at *k*_∣∣_ ≈ 4.5 μm^−1^ (see Fig. 3c). As thermalisation favours low energy states, the massively enhanced high energy emission contradicts thermalisation as main source of emission at *k*_∣∣_ = 0. To model this behaviour, we further leveraged the Green’s function approach (see ‘Methods’) for calculating the PL emission spectra using the random source approximation ^40^ (see SI). In the calculation, we used the momentum distribution of the excitons PL as a fitting parameter to fit the experimentally measured PL spectra. Our model, which shows good agreement with our experimentally obtained data (see Fig. 3b, c) strongly suggests that diffraction is the main source of the polariton PL at *k*_||_ = 0, as schematically shown in Fig. 2a. In particular, polaritons contribute to PL emission within the light cone due to the folding of the Brillouine zone of the metasurface, instead of thermalisation, leading to the observed PL enhancement. The excitons, however, can only relax into the light cone via thermalisation, i.e., inelastic scattering with phonons, which is less efficient. Our findings indicate that this PL mechanism is applicable to polaritons in periodic photonic structures, in general, where diffraction into the light cone can outpace energy relaxation to low momenta states, boosting their quantum yield. The additional low-energy peaks in the experimentally measured spectrum (Fig. 3c) arise from the charged and multi-body complexes, as described above. As these excitonic species do not possess sufficient oscillator strength to coherently couple to the resonant photonic mode in the metasurface, they are not accounted for in the theory.

### Optical control of spin configuration

**Fig. 4:**
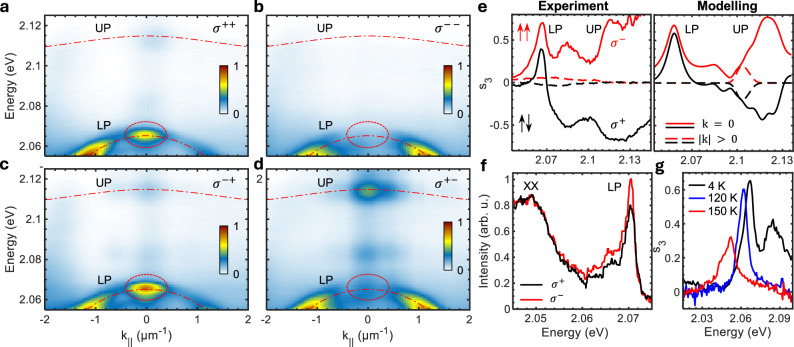
Angle- and polarisation-resolved PL spectra. **a**–**d** Circularly polarised components of the angle-resolved PL spectra of the sample excited with a circularly polarised laser pump. The *σ* ^*pe*^ convention reflects the sign of the circular polarisation of the pump (*p*) and the PL emission (*e*). **e** Measured and modelled s_3_ spectra with (black) *σ*^−^ and (red) *σ*^+^ polarised pump. The arrows (*↑*,*↓*) mark the spin configurations of the lower and upper polaritons, respectively, under (red) *σ*^−^ and (black) *σ*^+^ polarised excitation. **f** PL spectrum of the LP with (black) *σ*^+^ and (red) *σ*^−^ polarised pump, with the PL spectra of the biexciton (XX) plotted as reference. **g** s_3_ spectra of the LP at (black) *T* = 4K and (red) *T* = 150K, respectively.

Finally, we investigate the polarisation properties of the polaritons by performing polarisation-resolved PL measurements. Figure 4a–d shows the circularly polarised components of the momentum-resolved PL spectra (*σ* ^*i*+^ in Fig. 4a, c and *σ* ^*i*−^ in Fig. 4b, d) upon excitation with *σ*^+^ (Fig. 4a, d) or *σ*^−^ (Fig. 4b, c) polarised light. Remarkably, at *k*_||_ = 0, the LP emission is *σ*^+^ polarised (Fig. 4a, c), following the behaviour of the chiral BIC as seen in Fig. 1d. The polarisation of the UP, however, is opposite to that of the pump, both in *σ*^+−^ and *σ*^−+^ configurations, which is reminiscent of the rotating dipole behaviour of the exciton. This is because the LP (∣*C*∣^2^ = 0.76) mostly inherits the properties of the chiral photonic BIC state, while the UP (∣*X*∣^2^ = 0.76) behaves more like a valley exciton. Consequently, the emitted light of the UP and LP have opposite(same) signs of circular polarisations at *k*_||_ = 0 under *σ*^+^(*σ*^−^) polarised excitation.

We further analyse the antisymmetric polarisation behaviour by calculating the *s*_3_ = (*I* ^+^ − *I* ^−^)/(*I* ^+^ + *I* ^−^) spectra for both circular polarisations (*σ*^+^ and *σ*^−^) of the pump (see Fig. 4e). Here, UP and LP reach values of *s*_3_ > +0.7 with *σ*^−^ polarised excitation, and *s*_3_ ≈ −0.7 for the UP and *s*_3_ ≈ +0.4 for the LP with *σ*^+^ polarised excitation. Interestingly, *s*_3_ completely vanishes at large momenta, where the emitting states are mostly excitonic. This strong suppression of *s*_3_ is likely a result of inelastic scattering processes required for relaxation/thermalisation of excitons into the light cone. Additional inhomogeneous broadening due to the holes of the metasurface leads to a much larger experimental linewidth compared to our calculations, where only homogeneous linewidth broadening is considered.

At *k*_||_ = 0, the strong enhancement of circular polarisation is well reproduced by our model, indicating that, in addition to boosting PL intensity, diffraction also strongly enhances polarisation of the polaritons by bypassing the inelastic scattering processes. In combination with the contribution from the intrinsically chiral BIC, it leads to the strong antisymmetric polarisation of the UP and LP, where changing the polarisation of the pump laser triggers switching between parallel and anti-parallel spin configurations of the two polariton states (see Fig. 1a).

### Matter induced properties

The role of the matter component (the valley exciton) in the chiral BIC-like lower polariton becomes evident when measuring the absolute intensity of its PL under circularly polarised excitation. We find that the quantum yield is the strongest when the spin of the valley exciton is aligned with the polarisation of the chiral BIC (see Fig. 4f). The reference spectrum produced by the uncoupled biexciton (XX)^33^, on the other hand, shows no enhancement. Further, the matter component of the LP enables effective interaction with phonons, as shown in the temperature-dependent measurements (see Fig. 4g and SI), where both the energy and degree of circular polarisation drop with increasing phonon energies. These results demonstrate that by strongly coupling chiral BIC photons with valley excitons, we successfully created an intrinsically chiral hybrid state that possesses valley physics and effectively interacts with the surrounding medium, further distinguishing our findings from previous investigations of weakly coupled excitons^5^. Finally, we estimate a lifetime of 500 fs for the intrinsically chiral lower polaritons based on the lifetime and power-dependent measurements presented in the SI.

## Discussion

By strongly coupling valley excitons with chiral photonic BICs, we have created a new type of chiral hybrid quasi-particle, whose two energy eigenstates can possess distinct spin configurations, either parallel (*↑↑*) or anti-parallel (*↑↓*), depending on the circular polarisation of the optical excitation source (see Fig. 1a). By combining the unique physics of a chiral photonic and a spin-valley electronic system, we therefore complemented the portfolio of spin-selective chiral states with a light-matter hybrid particle with optically controllable spin properties. The potential for ultrafast optical switching between *↑↑* and *↑↓* polarised emission in response to circularly polarised light can be useful for spintronics, spin-based transistors, and chiral sensors.

## Methods

### Sample fabrication

To make the metasurface, we deposited a 220 nm TiO_2_ layer on a glass (SiO_2_) substrate by electron beam evaporation (0.065 nm/s). The TiO_2_ layer was then patterned using electron-beam lithography. This was done by depositing 20 nm of Cr and spin-coating a 80 nm thick film of PMMA. The PMMA was patterned using electron beam lithography, and the pattern then etched into the Cr layer with an inductively coupled Cl_2_ and O_2_ plasma. Further, we etched the TiO_2_ layer in a slant-etching system (as developed in ref. ^4^) with reactive O_2_, SF_6_, Ar and CHF_3_ ions. With this process, we created the slant-perturbed TiO_2_ metasurface consisting of a square array of trapezoid nanoholes, with the parameters: unit cell size *a* = 340 nm, width of hole *w* = 210 nm, height of hole *h* = 220 nm, slant angle *ϕ* = 0.1 and in-plane deformation angle *α* = 0.12, as presented in ref. ^4^ and schematically shown in Fig. 1b. The monolayer WS_2_ was mechanically exfoliated from a bulk WS_2_ crystal (sourced from HQ graphene) onto a gel-film (Gel Pak), and transferred onto the metasurface using the dry transfer technique.

### Theory

The reflectivity of the sample was calculated using the rigorous coupled wave analysis (RCWA)^41^. The RCWA method allows us to calculate both the angular and frequency spectra of the polarisation-resolved reflection coefficient and the total scattering matrix ${S}^{{{{\bf{QQ}}}}^{{\prime} }}$ connecting the multiple diffraction orders of the incoming and outgoing waves ${{{\bf{E}}}}^{{{\bf{Q}}}}={S}^{{{{\bf{QQ}}}}^{{\prime} }}{{{\bf{E}}}}^{{{{\bf{Q}}}}^{{\prime} }}$, where **Q** index labels different diffraction orders. The scattering matrix is also used to calculate the dyadic Green’s function of the metasurface, which we employ for calculating the PL emission spectra of our structure (see SI).

### Experimental setup

The PL and reflectivity measurements were conducted in a closed-cycle helium cryostat (Montana Instruments s50). For the PL measurements, the sample was excited with a 561 nm wavelength CW diode-pumped solid-state laser at a power of 30 μW. The source for the reflectivity measurements was a stabilised tungsten-halogen white light source. The laser or white light was focused with a high-NA objective (100×/0.9 NA) onto the sample surface, creating a light spot size of 0.45 μm in the focus of the objective. For larger area illumination, the laser or white light was focused into the back focal plane of the objective.   The reflected or emitted light from our structure was separated from the incoming light by a non-polarising 30:70 beam splitter cube, and collected with an array of lenses in a 4-f arrangement, where the back focal plane was focused onto the spectrometer slit of an Andor Shamrock i750 spectrograph, equipped with an Andor iXon 897 Ultra EMCCD camera, for imaging the presented momentum-resolved spectra. By employing a spatial filter in the real space image of our 4f-setup, we ensured that only the light stemming from the marked areas in Fig. 1b was collected and analysed. The presented reflectivity data were obtained by differential reflectance spectroscopy Δ*R*/*R*_*Ref*_ = (*R* − *R*_*Ref*_)/*R*_*Ref*_, using the reflectivity spectrum of the TiO_2_-coated glass surface next to the metasurface as a reference (Ref). For the polarisation-resolved measurements, we prepared the polarisation of the excitation beam with a linear polariser and quarter-wave phase plate. The polarisation of the detected light was analysed by a combination of super-achromatic quarter-wave phase plate and a linear polariser. To increase the signal-to-noise ratio in the presented polarisation-resolved images (Fig. 4a–d), we performed a moving average calculation in momentum space, effectively applying the average of 9 neighbouring pixels along *k*_∣∣_ to each pixel.

## Supplementary information

Supplementary Information

Transparent Peer Review file

## Data Availability

All data that support the findings of this study are available from the corresponding author upon request. The experimental data presented in the manuscript figures are available on figshare (https://figshare.com/s/c49d82ec72ca35c4b9c9).

## Code availability

The RCWA implementation is based on the publicly available code (https://github.com/jonschlipf/RigorousCoupledWaveAnalysis.jl). The simulated PL data were obtained by numerically evaluating Eq. (13) in the Supplementary Information. The corresponding source code is available from the authors upon request.
