# Supplementary material for: Intrinsically chiral exciton polaritons in an atomically-thin semiconductor
Source: Nat Commun. 2026 Mar 23;17:2742. doi: 10.1038/s41467-026-70875-5 (PMC13013978; doi:10.1038/s41467-026-70875-5)
Supplement: Supplementary file 1 — Supplementary Information [file 41467_2026_70875_MOESM1_ESM.pdf]

Supplementary Information
Intrinsically chiral exciton polaritons in an atomically-thin semiconductor

M. J. Wurdack,[1, 2, 3, 4, 5, *] I. Iorsh,[6, *] S. Vavreckova,[2, 3, 4] T. Bucher,[1, 3, 4] M. Król,[2] Z. Fedorova,[1, 3, 4] E. Estrecho,[2] D. Ilin,[7] S. Klimmer,[1, 4] L. P. L. Mawlong,[8] H. Deng,[9] Q. Song,[9] T. van der Laan,[8] G. Soavi,[1, 4] T. Pertsch,[3, 4] F. Eilenberger,[3, 4, 10] I. Staude,[1, 3, 4] Y. Kivshar,[11] and E. A. Ostrovskaya[2]

[1] *Institute of Solid State Physics, Friedrich Schiller University Jena, 07743 Jena, Germany.*
[2] *Department of Quantum Science and Technology, Research School of Physics, The Australian National University, Canberra, ACT 2601, Australia*
[3] *Institute of Applied Physics, Friedrich Schiller University Jena, 07745 Jena, Germany.*
[4] *Abbe Center of Photonics, Friedrich Schiller University Jena, 07745 Jena, Germany.*
[5] *Department of Chemical Engineering, Stanford University, Stanford, CA, USA*
[6] *Department of Physics, Engineering Physics and Astronomy, Queen's University, Kingston, Ontario, K7L 3N6, Canada.*
[7] *School of Mathematical and Physical Sciences, University of Technology Sydney, Ultimo, NSW 2007, Australia*
[8] *Manufacturing, CSIRO, West Lindfield, Sydney, NSW, 2070 Australia.*
[9] *Harbin Institute of Technology, Shenzhen 518055, China.*
[10] *Fraunhofer Institute for Applied Optics and Precision Engineering IOF, Albert-Einstein-Str. 7, 07745 Jena, Germany*
[11] *Nonlinear Physics Center, Research School of Physics, Australian National University, Canberra ACT 2601, Australia.*

Sample Design and Characterisation: A schematic illustration, SEM image, and AFM image of the bare slant-perturbed TiO_2 metasurface and metasurface/$WS2_2$ heterostructure are shown in Fig. 1a-c, respectively. As shown in the images, the metasurface consists of a square array of trapezoid nanoholes, with designed unit cell size $a = 340$ nm, hole width $w = 210$ nm, hole height $h = 220$ nm, slant angle $\phi = 0.1$ and in-plane deformation angle $\alpha = 0.12$, as discussed more in detail in [1]. The monolayer was mechanically exfoliated and transferred onto the metasurface using the dry transfer method. The AFM and SEM images show that the fabricated structure is consistent with the design, and that the arrangement and geometries of the holes are regular.

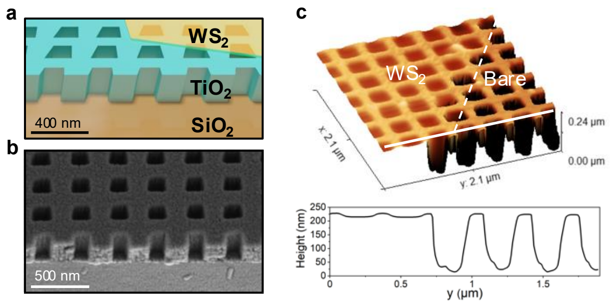

FIG. 1. Design of the hybrid metasurface/2D semiconductor heterostructure. **a** Schematic illustration of the heterostructure. **b** Scanning electron microscope (SEM) image of the metasurace. **c** Atomic force microsce (AFM) image of the heterostructure. The interface between metasurface/WS_2 and bare metasurface is marked with a dashed white line, and the position of the (bottom) line profile with a solid white line.

To evaluate local fluctuations of the photonic reso-

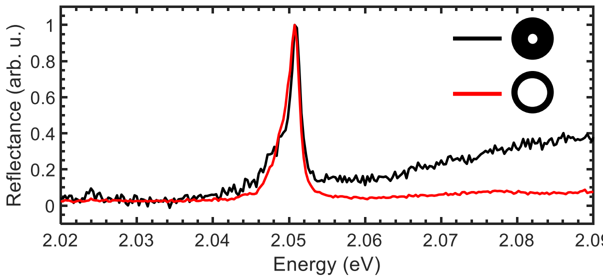

FIG. 2. High resolution spectra of the chiral photonic resonance averaged over an area of (black) ~ 400 μm and (red) ~ 2000 μm^2.

nance on the spatial scales relevant to our monolayer size, we compared the reflectivity spectrum of the chiral mode averaged over an area smaller than the monolayer (~ 400 μm^2) to that of a larger area ($\sim 2,000 \mu m^2$), consisting of $\sim 4,000$ and $\sim 20,000$ holes, respectively. This experiment was done by employing a spatial filter in the real space plane of our experimental setup and extracting the momentum-space spectrum of the σ^+ component of the reflected white light at $k = 0$. Figure 2 shows the high-resolution spectra of the chiral resonance for both configurations. When comparing the linewidths, we can observe that local fluctuations of the hole geometries across the metasurface lead to the inhomogeneous broadening of the resonance. However, since the amount of broadening is only in the order of 1 meV, which is an order of magnitude smaller than the inhomogeneous broadening of the excitons (see main text), we conclude that local fluctuations are relatively small and that the spatial geometries of the holes are sufficiently consistent for producing a high-Q resonance across the whole mono-

layer area.

Theory: The metasurface is a structure with discrete translational invariance. The in-plane component of the wavevector is conserved modulo reciprocal lattice vectors $\mathbf{Q}$. In the systems with continuous translational invariance we can express the electric field vector $\mathbf{E_k}$ as

$$\mathbf{E_k}(z) = \int dz' G_{\mathbf{k}}(z,z')\mathbf{P_k}(z'), \quad (1)$$

where $\mathbf{k}$ is the in-plane wavevector, $\mathbf{P}$ is the polarisation at a specific wavevector and specific position along the z axis. In the system with discrete translational invariance we instead should write

$$\mathbf{E_{k+Q}}(z) = \int dz' G_{\mathbf{k}}^{\mathbf{Q},\mathbf{Q}'}(z,z')\mathbf{P_{k+Q'}}(z'), \quad (2)$$

where $\mathbf{Q},\mathbf{Q}'$ span over all the reciprocal lattice vectors and the summation is taken over the repeating indices $\mathbf{Q}'$. For each specific values of $\mathbf{k}, z, z'$, G is the matrix consisting of 3×3 blocks labeled by $\mathbf{Q}$ and $\mathbf{Q}'$ which connect the 3 electric field components with momenta $\mathbf{k}+\mathbf{Q}$ and at position z to the polarisation with momenta $\mathbf{k}+\mathbf{Q}'$ at position z'. In our analysis we will only consider in-plane polarisation induced by excitons and model in-plane components of the emitted electric field, therefore we will only consider 2×2 blocks.

We assume that the monolayer is placed at the $z=0$ plane and that both incident and emitted radiation is in the $z>0$ half space. For further analysis we will need the Green's functions corresponding to $z=z'=0$ and to $z\to\infty, z'=0$. While the former connects the polarisation at the plane of the monolayer to the electric field at the same plane, the latter connects the monolayer polarisation to the far field which is ultimately detected in the experiment.

The Green's function can be expressed via the reflection matrix $\hat{r}$:

$$G_{\mathbf{k}}^{\mathbf{QQ}'}(0,0) = [\hat{I}+\hat{r}]\cdot[\delta_{\mathbf{QQ}'}\otimes G_{0,\mathbf{k+Q}}], \quad (3)$$

where $\hat{I}$ is the identity matrix, G_0 is the Green's function for the free space:

$$G_{0,\mathbf{k+Q}} = \frac{i}{2k_0^2k_z}\begin{pmatrix} k_0^2-k_x^2 & -k_xk_y \\ -k_xk_y & k_0^2-k_y^2\end{pmatrix}, \quad (4)$$

and $\hat{r}$ is the reflection consisting of 2×2 blocks

$$\hat{r}^{\mathbf{QQ}'} = \begin{pmatrix} r_{xx}^{\mathbf{QQ}'} & r_{xy}^{\mathbf{QQ}'} \\ r_{yx}^{\mathbf{QQ}'} & r_{yy}^{\mathbf{QQ}'}\end{pmatrix} \quad (5)$$

The reflection matrix is computed numerically using the rigorous coupled wave analysis (RCWA) [2]. We note that RCWA allows us to calculate the generalised transfer matrices in the whole structure, which in turn can be used to restore the local field distributions. The electric field distribution at the wavelength of the chiral BIC $\lambda \approx= 597$ nm maps in three orthogonal planes are shown in Fig. 3.

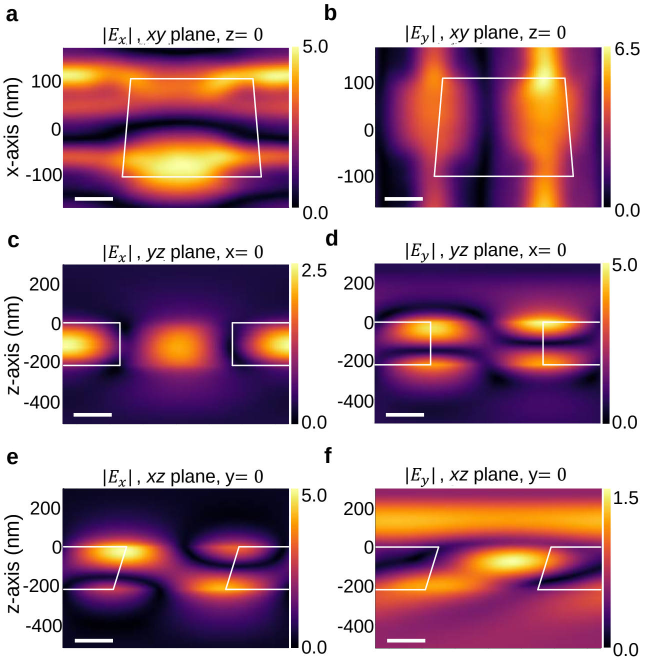

FIG. 3. **Electric field distribution of the BIC mode at $\lambda = 597$ nm. a,b** E_x and E_y in the xy-plane at the top surface, where we place our monolyer (z=0), respectively. **c-d** E_x and E_y components of the cross-sections in the (c-d) yz- and (e-f) xz-planes, at x=0 or y=0, respectively. The contour of the metasurface is marked as white solid lines. The scale bar size is 50 nm.

The polarisation of excitons $\mathbf{P_q}(z,t) = \mathbf{P_q}(t)\delta(z)$ satisfies the equation:

$$\dot{\mathbf{P}}_{\mathbf{q}}(t) = (-i\omega_X(\mathbf{q}) - \Gamma - i\frac{1}{2}\mathbf{\Omega_q}\sigma)\mathbf{P_q} - \quad (6)$$
$$\gamma\mathbf{E_q}(z=0,t) + \chi_{\mathbf{q}}(t), \quad (7)$$

where ω_X, Γ are the frequency and non-radiative decay rates of excitons, $\mathbf{\Omega_q}$ is the effective exciton magnetic field due to the TE-TM exciton splitting, γ is the excitonic radiative decay rate, and χ is the pumping rate, i.e. the rate of exciton injection from the reservoir. The pumping rate χ is modeled within the random source model [3] as a random process satisfying

$$\langle\chi_\alpha(\mathbf{q},t)\rangle = 0, \quad (8)$$
$$\langle\chi_\alpha^*(\mathbf{q},t)\chi_\beta(\mathbf{q}',t')\rangle = \rho_{\alpha\beta}(\mathbf{q},t)\delta_{\mathbf{q},\mathbf{q}'}\delta(t-t') \quad (9)$$

where ρ_{ij} is defined both by the polarisation of the pump and by the relaxation kinetics of excitons due to exciton-phonon and exciton-exciton interactions.

The equation (7) for $\mathbf{P}$ can be solved exactly, and then $\mathbf{P}$ can be substituted into Eq. (2), which results in the equation for the total field at $z=0$ which again can be solved exactly. Finally, we obtain the far-field expression for the electric field:

$$E_\alpha(\mathbf{k}, z \to \infty, \omega) = \quad (10)$$

$$G^{0\mathbf{Q}}_{\mathbf{k},\alpha\beta}(-\infty, 0, \omega)[\hat{\alpha}_0(\mathbf{k}+\mathbf{Q}, \omega)^{-1} \quad (11)$$

$$\delta_{\mathbf{QQ}'} - \hat{G}_{\mathbf{k}}(0,0,\omega)]^{-1,\mathbf{QQ}'}_{\beta\xi} \chi^{\mathbf{Q}'}_{\xi}/\gamma, \quad (12)$$

where α_0 is the bare exciton polarisability, $\alpha_0^{-1}(\mathbf{q},\omega) = (\omega - \omega_X(\mathbf{q}) + i\Gamma - \frac{1}{2}\mathbf{\Omega}_\mathbf{q}\sigma$. We note that only the zeroth diffraction order is accounted for in the far field, since the period of the structure is less than the wavelength. From Eq. (12) it can be seen that the eigenfrequencies and eigenmodes of the whole structure are given by the condition Det $\left[\hat{\alpha}_0(\mathbf{k}+\mathbf{Q},\omega)^{-1} - \hat{G}_{\mathbf{k}}(0,0,\omega)\right] = 0$, and the profile of the polariton mode can be restored from the kernel vector of this matrix. The kernel vector has two components of in-plane electric field for each diffraction order $\mathbf{Q}$. Therefore, we can image a polariton mode profile at specific $\mathbf{k}$ as an image on the reciprocal lattice where each node depicts a polarisation ellipse for a specific diffraction order. These plots are shown in Figs. 4(a,b) for the lower polariton mode at $\mathbf{k} = 0$. The red and blue lines correspond to the two circular polarisations (helicities), while the violet color depicts linear polarisation. We can see that the helicity of the polariton near field η satisfies $\eta(\mathbf{Q}) = -\eta(-\mathbf{Q})$ due to the time reversal symmetry. We also note that while for the achiral structure (without the slant) the main diffraction orders are linearly polarised [Figs. 4(b)], for the chiral structure two main diffraction orders along y axes have finite and opposite helicities [Figs. 4(a)]. The slant angle leads to the asymmetric scattering of $+1$ and -1 diffraction orders to the far field, which ultimately leads to the circularly polarised far-field emission.

We can calculate the far-field intensity from the electric field and average it over the random sources obtaining the final expression for the polarisation resolved PL intensity $I_{\alpha,\alpha'}$

$$I_{\alpha'\alpha}(\mathbf{k}) = \gamma^{-2} R^{*\mathbf{Q}'}_{\alpha'\beta'} \rho_{\beta',\beta}(\mathbf{k}+\mathbf{Q}',\omega) R^{\mathbf{Q}'}_{\alpha\beta}; \quad (13)$$

$$R^{\mathbf{Q}'}_{\alpha\beta} = G^{0\mathbf{Q}}_{\alpha\xi}(\hat{\alpha}_0^{-1} - \hat{G})^{-1,\mathbf{QQ}'}_{\xi\beta} \quad (14)$$

Equation (14) relates the far-field emission intensity to the stationary distribution of the exciton pump rate ρ. We note that this distribution is qualitatively different from the thermal distribution since the system is out of equilibrium due to pump and radiative decay. Moreover, since the polaritons have negative effective mass, there can be no thermal equilibrium in the system. While precise determination of the distribution function would require the solution of a master equation for the density matrix accounting for the exciton-phonon and exciton-exciton scattering, we can adopt a generic expression for $\rho_{\alpha\beta}$:

$$\rho_{\alpha\beta}(\mathbf{q},\omega) = \rho^{(0)}_{\alpha\beta}(1 - e^{-q^2/\tilde{q}^2}), \quad (15)$$

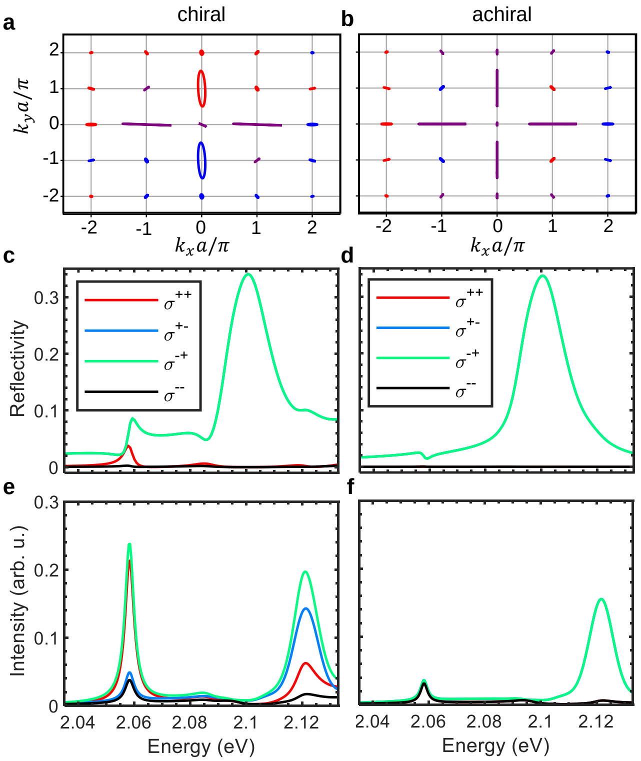

FIG. 4. **Modeled polarisation properties, and reflectivity and PL spectra of exciton-polaritons in (with slant) chiral and (without slant) achiral geometries. a,b** Polarisation of the lower polariton in the diffractions orders, with (blue) σ^-, (red) σ^+ and (purple) linear polarisation, and the ellipsity corresponding to the degree of circular polarisation, for chiral and achiral structures, respectively. **c,d** Polarisation resolved reflectivity spectra of the modeled chiral and achiral structures, respectively, where σ^{ir} convention indicates polarisations of the incoming (i) and reflected (r) light. **e,f** Polarisation resolved PL spectra of the modeled chiral and achiral structures, respectively, where σ^{pe} convention indicates polarisations of the pump (p) and emitted light (e).

where $\rho^{(0)}$ is the polarisation matrix of the optical pump, and $\tilde{q}$ is the fitting parameter. The dependence in Eq. (15) reflects the suppression of the pump to $q = 0$ states and a relatively stronger pump to the high q states lying outside the light cone.

The calculated reflectivity and PL spectra based on our model for chiral (with the slant) and achiral (without the slant) geometries are presented in $4c - f$. The different underlying physics between reflectivity and PL spectra are seen in both cases, where the exciton response dominates the reflectivity spectrum, while the polariton emission dominates the PL spectrum. This shows that the intensity enhancement of the polariton PL is mainly due to the Brillouin zone folding. This effect overcomes the thermalisation process, as discussed in the main text, and therefore is independent on chirality. However, as seen for the lower polariton, the chiral geometry induces

asymmetry between the spectra produced by σ^+ and σ^- polarised white and laser light, respectively. This behavior underlines the intrinsically chiral nature of the exciton polaritons forming in the regime of strong coupling between chiral BIC photons and excitons.

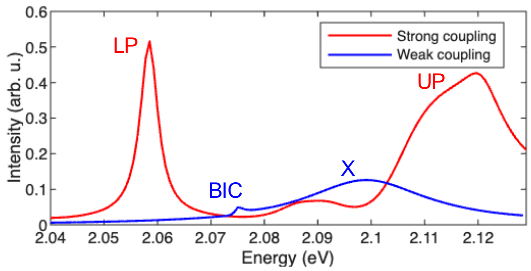

FIG. 5. **Calculated absolute PL intensities at $k = 0$ for weakly and strongly coupled systems.** The lower (LP) and upper (UP) polariton peaks in the strong coupling regime are shown in red and the chiral BIC and exciton (X) peaks in the weak coupling regime are shown in blue.

To estimate the effects of strong coupling on the total PL intensity, we compare the calculated PL spectrum with that of a comparable system in the weak coupling regime. For the latter, we added 100 nm spacing between the monolayer and the metasurface, which substantially inhibited energy exchange interaction between the excitons and photons. The resulting spectra are shown in Figure 5. In addition to the two-level repulsion, strong coupling significantly enhances overall PL emission from both the intrinsically chiral (lower polariton) state and the more exciton-like upper polariton state, as discussed in the main text.

Experiment: To probe polaritons and excitons in our system, we performed polarisation resolved PL and reflectivity measurements at normal incidence (see Fig. 6a-d)). Therefore, we employed either a circularly polarised cw-laser ($\lambda = 561nm$) or circularly polarised white light. For σ^+ polarised light sources (see Fig. 6a,b), the σ^+ polarised component of the emitted and reflected light yields a strong lower polariton peak. The σ^- component, on the contrary, stems mainly from the upper polariton in the PL and from the exciton in the reflectivity measurement. This behavior changes dramatically for σ^- polarised light sources (see Fig. 6c,d), where contributions of excitons and polaritons are all strongest for the σ^+ polarised component of the emitted and reflected light, and suppressed for the σ^- polarised component. This results from the interplay between intrinsically chiral states and rotating dipoles, combined with diffraction induced photoluminescence, as discussed in the main text. As the lower polariton possess robust σ^+-polarisation, independent on the polaritons of the pump, and is only receptive to σ^+-polarised whitelight, we conclude that the lower polariton is an intrinsically chiral light-matter hybrid state, inheriting the physics from the intrinsically chiral

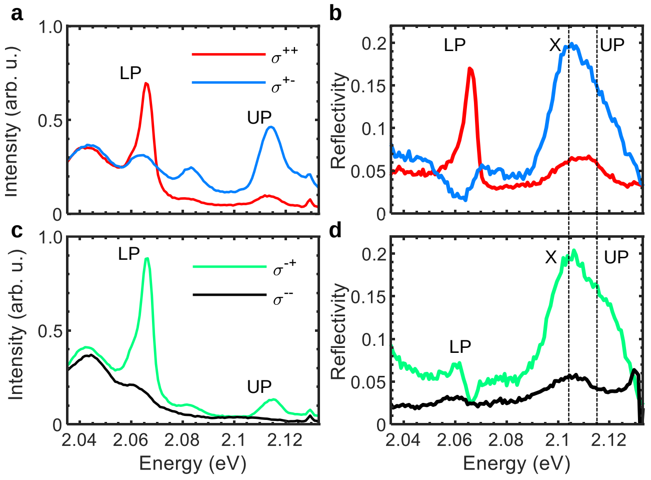

FIG. 6. **Polarisation resolved reflectivity and PL spectra of our structure. a,b** Polarisation resolved photoluminescence and reflectivity spectra at $k = 0$ using σ^+ polarised laser excitation or white light, respectively. **c,d** Polarization resolved photoluminescence and reflectivity spectra at $k = 0$ using σ^- polarised laser excitation or white light, respectively.

photonic BIC [1]. As the polaritons have a matter (exciton) component, we also expect direct interactions with the environment, e.g., with phonons, which we probe by performing temperature dependent measurements.

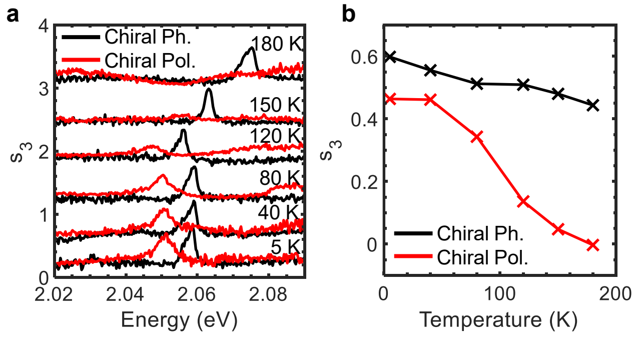

FIG. 7. Temperature dependent measurements of the degree of circular polarisation. **a**. Waterfall plot of the s_3 spectra at $k = 0$ for (black) chiral photons in the bare metasurface and (red) polariton PL on the monolayer for various temperatures between 5 and 180 K. **b** Peak amplitudes of the s_3 spectra shown in panel (a) as a function of temperature.

As shown in Fig. 4g of the main text, the energy and s_3 value of the lower polariton decreases, and the linewidth increases with temperature. These trends can be explained by the stronger interaction with phonons at higher temperatures, leading to depolarisation, reduction of lifetime, and energy exchange. We test the robustness of our observation further by fabricating another, similar structure, and repeating the reflectivity and PL measurements on the bare metasurface and the metasurface/WS_2

heterostructure, respectively. Figure 7a,b shows the s_3 spectra and peak s_3 values of the chiral photons and polaritons for a larger temperature range. In this sample, the chiral polariton PL diminishes at temperatures above 120 K, likely because of doping and less robust strong light-matter coupling compared to the sample discussed in the main text. However, between 5 K and 120 K, we observe polariton redshift and linewidth broadening with increasing temperature, similar to what we have observed in the sample discussed in the main text. Most strikingly, phonon-induced depolarisation of the chiral polariton causes a sharp drop of s_3 with temperature, while the photonic resonance in the metasurface remains chiral (see Fig. 7b). Therefore, phonon-induced depolarisation and linewidth broadening is much stronger pronounced for the polariton compared to the photon, which can be explained by dominating temperature dependent phonon-polariton interactions via the excitonic component of polaritons.

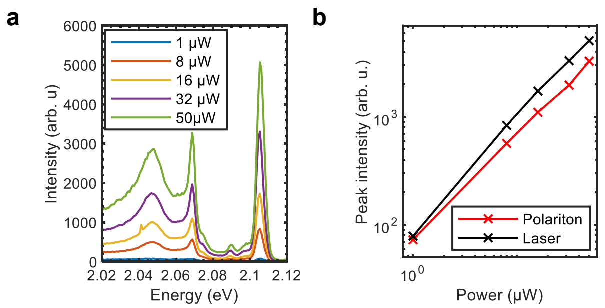

FIG. 8. **Power dependent measurements of the lower polariton.** **a** Power dependent PL spectra of lower polaritons at $k = 0$ (see text). **b** Peak intensities of (black) the filtered laser at 2.13 eV and (red) the lower polariton.

To verify that our polaritons are in the thermal regime and behave like single particles, we performed power dependent measurements with 200 fs laser pulses tuned resonantly to the upper polariton state (see Fig. 8). To suppress the pulsed laser peak in the measured spectra, we excited with horisontally polarised light, and measured only vertically polarised light in the detection path, using linear polarisers. When extracting the peak intensities for the lower polariton peak at ~ 2.07 eV in Fig. 8b, we can detect a slightly sub-linear input-output characteristics for the polaritons in comparison to the pump laser. Thus, the polaritons exhibit no nonlinear behavior and behave like single particles. This confirms that the polaritons are in the thermal regime within the shown range of average powers, and we can estimate their lifetime and temporal coherence from the linewidth measurement [4].

To estimate the lifetime, we measured a high resolution spectrum of the chiral lower polariton, as presented in Fig. 9. By fitting a Voigt profile to the spectrum, we can retrieve both homogeneous and inhomogeneous linewidth broadening (ΔE^H and ΔE^{IH}, respectively), whereas homogeneous broadening scales inversely with the lifetime. Thus, with the fitted linewidths of $\Delta E^H \approx 2.5$ meV and $\Delta E^{IH} \approx 0.2$ meV, the polaritons have an estimated lifetime of $\tau = \hbar/\Delta E \approx 530$ fs, which is consistent with the coherence times previously determined for confined WS_2 polariton states with low inhomogeneous broadening [4].

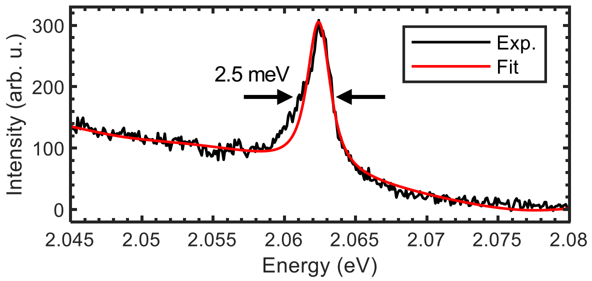

FIG. 9. High-resolution spectrum of the PL at $k = 0$, with the chiral lower polariton state fitted with a Voigt profile.

* These authors contributed equally.

[1] Y. Chen, H. Deng, X. Sha, W. Chen, R. Wang, Y.-H. Chen, J. C. Dong Wu, Y. S. Kivshar, S. Xiao, and C.-W. Qiu, Observation of intrinsic chiral bound states in the continuum, Nature **613**, 474–478 (2023).

[2] J. Schlipf and I. A. Fischer, Rigorous coupled-wave analysis of a multi-layered plasmonic integrated refractive index sensor, Optics Express **29**, 36201 (2021).

[3] L. Deych, M. Erementchouk, A. Lisyansky, E. Ivchenko, and M. Voronov, Exciton luminescence in one-dimensional resonant photonic crystals: A phenomenological approach, Physical Review B—Condensed Matter and Materials Physics **76**, 075350 (2007).

[4] M. Wurdack, E. Estrecho, S. Todd, T. Yun, M. Pieczarka, S. K. Earl, J. A. Davis, C. Schneider, A. G. Truscott, and E. A. Ostrovskaya, Motional narrowing, ballistic transport, and trapping of room-temperature exciton polaritons in an atomically-thin semiconductor, Nature Communications **12**, 5366 (2021).
