# Supplementary material for: Intrinsically chiral exciton polaritons in an atomically-thin semiconductor
Source: Nat Commun. 2026 Mar 23;17:2742. doi: 10.1038/s41467-026-70875-5 (PMC13013978; doi:10.1038/s41467-026-70875-5)
Supplement: Supplementary file 2 — Transparent Peer Review file [file 41467_2026_70875_MOESM2_ESM.pdf]

nature portfolio

Peer Review File

Intrinsically chiral exciton polaritons in an atomically-thin semiconductor

Corresponding Author: Dr Matthias Wurdack

This manuscript has been previously reviewed at another journal. This document only contains information relating to versions considered at Nature Communications.

This file contains all reviewer reports in order by version, followed by all author rebuttals in order by version.

Version 0:

Reviewer comments:

Reviewer #3

(Remarks to the Author)
I am satisfied with the response of the reviewers. The experimental data is still weak in my opinion. However, given the overall quality of the work, I recommend this for publication in Nature Communications.

Open Access This Peer Review File is licensed under a Creative Commons Attribution 4.0 International License, which permits use, sharing, adaptation, distribution and reproduction in any medium or format, as long as you give appropriate credit to the original author(s) and the source, provide a link to the Creative Commons license, and indicate if changes were made.
In cases where reviewers are anonymous, credit should be given to 'Anonymous Referee' and the source.
The images or other third party material in this Peer Review File are included in the article's Creative Commons license, unless indicated otherwise in a credit line to the material. If material is not included in the article's Creative Commons license and your intended use is not permitted by statutory regulation or exceeds the permitted use, you will need to obtain

permission directly from the copyright holder.
To view a copy of this license, visit https://creativecommons.org/licenses/by/4.0/

Response to Reviewers:

Reviewer #3

Reviewer’s comment:
I am satisfied with the response of the reviewers. The experimental data is still weak in my opinion. However, given the overall quality of the work, I recommend this for publication in Nature Communications.
Our response:
We thank the Reviewer for the positive assessment of our work and for recommending it for publication in Nature Communications.
